# The Role of Personality Traits through Habit and Intention on Determining Future Preferences of Public Transport Use

**DOI:** 10.3390/bs7010008

**Published:** 2017-02-17

**Authors:** Mahdi Yazdanpanah, Mansour Hadji Hosseinlou

**Affiliations:** 1 Transportation Engineering, K. N. Toosi University of Technology, Tehran 1996715433, Iran; 2 Department of Civil Engineering, K. N. Toosi University of Technology, Tehran 1996715433, Iran; mansour@kntu.ac.ir

**Keywords:** public transport use, sustainable transportation, personality traits, intention, habit, car access, structural equation modeling

## Abstract

A complex set of factors may affect transportation mode choice. While earlier studies have often considered objective factors in determining preferences of public transport use as a sustainable transportation, subjective factors such as personality traits are underexplored. Therefore, this study aimed to investigate the influence of personality traits on the number of future public transport use. Additionally, “car habit” and “intention toward using public modes” were considered to be important. For this purpose, a case study from departure passengers at Imam Khomeini International Airport (IKIA, Tehran, Iran) was conducted between January and February 2015 at IKIA. Results of structural equation modeling (SEM) shows that only neuroticism and extraversion personality traits were significant in determining future public transportation mode choice. However, the model indicates that these traits indirectly influence intention and car habit. Neuroticism was found to have a total effect of −0.022 on future public transport use, which represents a negative association with public transport use, while extraversion positively influenced future public transport use with a total effect of 0.031. Moreover, the results found interestingly that car access had a better fit to the data than the number of cars in household (NCH); both had significant positive effect on car habit, but only car access had a significant influence on intention. Furthermore, the effect of socio-demographic variables such as age, gender, educational level, income level, and body mass index (BMI) were determined to be significant in identifying choice of future transport mode to airports, which is explained in the discussion section of this paper.

## 1. Introduction

Previously, objective variables such as travel time and cost have been widely used for determining the transportation mode choice process. However, in spite of the significance of some qualitative variables such as comfort, convenience, safety, and reliability outlined in numerous transportation mode choice studies, there is no standard measure for measuring these variables [1].

Personality traits and their impacts on various behavioral patterns have been investigated in previous studies [2,3,4,5,6,7,8,9]. Specifically, in transportation studies, there is considerable literature about personality traits and their relationship with driving style and risk-taking behaviors [10,11,12,13,14,15,16,17,18,19,20,21]. Almost all of these studies conclude that assessing personality traits is useful for predicting driving behavior. However, there have been limited studies that investigate their roles in determination of transportation mode choice. This could be attributed to resources required for understanding their effects [1] which involve adding these psychological variables to the survey, which makes it long, along with the concern that they may not be easily forecasted [22]. Few studies have specifically considered the effect of personality traits on transportation mode choice. Results of these studies demonstrate the effect of personality traits on the use of priced managed lanes and indicated that conscientious individuals have less preference for carpooling on managed lanes [23]. Investigating the relation between personality traits and travel behavior indicated that extraverted individuals had more willingness to travel longer distances compared with introverted individuals [24]. Moreover, there was an attempt using a 16PF psychometric test to assess the effect of personality traits on transportation mode choice [25].

On the other hand, habits play a substantial effect on shaping one’s behavior and are widely considered for prediction and explanation of human behavior [26]. Travel behavior is often regarded as habitual [27], and habits play a crucial role in transportation behavior [28,29,30,31]; therefore, habit was determined to be a significant predictor of transportation mode choice [32]. However, changing habitual travel behavior may be difficult to implement [33] and synchronic habits can significantly affect urban transportation behavior of individuals [34]. Investigating the impact of habit and car access on student behavior uncovered that habit was the strongest factor that influences behavior, while car access only significantly influenced habit rather than having a direct effect on actual behavior [35].

Additionally, theories dealing with human decision process such as the Theory of Planned Behavior (TPB) indicate that behavior is influenced by intention [36]. Earlier studies have found significant interactions between habit and intention; when habit was weak, the intention was significantly related to behavior [37], which is consistent with a study of the relation between active commuting and habit strength. It has been demonstrated that stronger active commuting habits weaken the association between intention and bicycle use [38]. It was found that car habit negatively predicts intention of public transportation usage and use of public transportation appears to partly reflect an arranged and deliberate psychological process [39].

To the best of our knowledge, no prior study has used personality traits in predicting future public transport usage as a sustainable mode. Additionally, the influence of car access and socio-demographic characteristics are examined in this study. Furthermore, the correlation of body mass index (BMI) with active travel such as walking/biking and use of public transportation has been examined in several studies, with most finding a connection between lower BMI or a healthy body weight and active travel [40,41,42,43,44,45,46]. That being said, however, other studies reported limited evidence of association between adult BMI and active transport [47] and none between public transport accessibility and obesity [48]. This study attempts to find the probable effect of individuals’ BMI on future public transport use.

Accordingly, it seeks to examine associations between personality traits, car habits, and future public-transport-usage intention by employing structural equation modeling (SEM) techniques. One application of SEM is path analysis, which can model complex relationships like those considered in this study. In addition, it must be noted that we avoid the use of latent variables to examine the model for forecasting purposes. For measuring personality, this paper uses the Big Five traits, also known as the five-factor model (FFM), due to the assumption that it encompasses “most generalizable, empirically rooted, and theoretically sound model of personality” [49]. In fact, after several decades of use, it has been found to be valid, reliable, and useful for different cultures [50]. The NEO Personality Inventory (NEO PI-R) is the questionnaire used to measure the five major personality domains. The validity of Persian NEO-PIR was assessed via self-ratings and peer-ratings of 200 students of Iranian culture; results showed that the Persian form of the questionnaire had acceptably high validity and reliability measures that were similar to those of the original instrument [51]. A shorter version of the questionnaire, the NEO Five-Factor Inventory (NEO-FFI), was reliability-tested through a study of 630 students of Iranian culture. The reliability study revealed that only the openness to experience domain did not have sufficient reliability; the Cronbach’s alpha equaled 0.39 [52].

It must be noted that cultural tradition may contribute to differences in individuals’ behaviors [53,54], and these differences may also be reflected in their travel patterns [55]. Hence, Iranian travelers, who might have specific traditions developed over centuries of cultural advancement, can show behaviors regarding transport mode use that are distinct from those of other cultures. Personality traits, habit and intention, which are the main variables in this paper, might be different across cultures [56]. For example, they may not be equally important [57] or they may be temporally situated [58]. Therefore, the results of this study may only represent the behaviors or tendencies of the Iranian people, and other studies would need to investigate whether cultural or temporally situated transport behavior of transport is based on the above-mentioned cognitive variables. Literature regarding psychological processes involved in transportation mode use in other countries may partly be valid in modern Iran because there are similarities in human behavior worldwide, but some specific behavior might be specific to Iran, as it is a religious country that is also transitioning into an industrial one.

This study attempts to propose new insights for understanding public transport usage as a means of a sustainable transport by including personality traits to the SEM model and by making an effort to understand the possibility of predicting public transport use by means of dimensions unlike the traditional objective factors such as travel time and cost. The main research questions are whether personality traits can help predict future public transport use and whether is it possible to understand differences in individual behavior regarding public modes of transportation by considering personality traits.

Based on previous studies that associated personality traits with several aspects of human behavior [2,3,4,5,6,7,8,9], it would be appealing to examine their efficacy in predicting transport mode use. The lack of studies that comprehensively consider personality traits based on the well-known big five-model of personality is evident. However, one recently published paper on the influence of personality traits on choice of public mode of transport [59] used a hybrid latent class discrete choice model to reveal the heterogeneities between personality traits in choosing public modes of transportation to the airport. However, in this study, transport behavior was examined mostly from a psychometric viewpoint rather than an econometric one.

The remainder of the paper is structured as follows. Section 2 contains the procedures, methods, and materials. Section 3 deals with results and Section 4 represents dissection of important findings, and Section 5 concludes the paper and offers some new avenues for future research.

## 2. Materials and Methods

### 2.1. Data Collection

#### 2.1.1. Sampling Method

A random systematic paper-based survey was used to collect data from departure passengers waiting at check-in lounge of Imam Khomeini International Airport (IKIA) during January and February 2015. Systematic random sampling, also known as sequential sampling, is a method in which every nth individual is selected from the population. In this method, the first respondent is selected randomly from among the first n individuals. Systematic sampling is recognized as a good technique for airport surveys and is considered to be equivalent to simple random sampling [60]. IKIA is the largest airport in Iran which currently serves only international flights with a total annual capacity of 6.5 million passengers [61]. The airport can be accessed by private car and taxi. Public modes such as bus and metro are not currently available. However, Metro Line 1 is currently under construction to reach this airport from the city of Tehran. After validating the responses by checking that the respondents answered the questions thoroughly, 557 completed surveys were analyzed in this study.

#### 2.1.2. Sample Characteristics

Iran is the 18th largest country in the world with an estimated 2015 population estimated at 80 million, which ranks 17th by population in the world [62]. The median age of Iran’s population is 29.4 years [62], and males comprise 51% [63]. Nearly one quarter (24.9%) of Iranian adults are obese [64]. The sample consisted of 110 respondents with a diploma (19.7%), 217 respondents with a bachelor degree (39%), 160 respondents with master degree (28.7%), and 70 respondents with Ph.D. (12.6%). Nearly two third of respondents were male (64.6%), and 197 respondents were female (35.4%). The average age of respondents was 37.9 (Std. Dev. = 11.72), which ranged from 17 to 75 years old. It consisted of 192 single (34.5%) and 365 married respondents (65.5%). The majority of respondents (95.5%) had at least one car in the household. Regarding monthly income, 177 (31.8%) received less than 30 million IRR, whereas nearly half of the respondents (45.6%) had a monthly income of 30–100 million IRR. Additionally, 49 respondents (8.8%) had an income range of 100–200 million IRR, and only 39 respondents (7%) had more than 200 million IRR in monthly income. In addition, 38 respondents (6.8%) did not disclose their income level. The average body mass index (BMI) of respondents was 24.7 (Std. Dev. = 3.98), which was obtained based on their stated weight and height.

#### 2.1.3. Sample Representativeness

Comparing the socio-economic characteristics of our sample with a previous study conducted among air passengers at IKIA [65], comprising 73.6% male and 67.2% married respondents, shows the similarity between the two studies in terms of demographic characteristics of the sample. For validating the gathered sample, data from previous studies conducted in 2011 indicated that the majority of respondents at the IKIA are male (75.8%) [66]. Another survey of Tehran air travelers revealed that 83.2% were male, and that 70.6% were university graduates [67], which is consistent with our study. Moreover, because IKIA only serves international destinations, respondents may have higher than average incomes, as evidenced by their ability to travel internationally. In addition, public transport to this airport currently does not exist, so in consideration of the above-mentioned survey data from air travelers of Tehran, it seems that this study’s data is much more representative of the air-traveler population than of the Iranian population as a whole.

### 2.2. Survey Measures

Personality traits were measured by NEO-FFI instrument which has 60 items under the “Big Five” traits [50]. Each trait was evaluated using 12 items and was measured using a 5-point Likert scale ranging from strongly disagree (0) to strongly agree (4). The Persian version of the NEO-FFI was acquired from one of the popular cognitive institutes of Iran that translates psychological instruments devised by many academics and well-known researchers. In addition to translation services, the institute provides information about the instrument validation and administration guidance. Therefore, the validation of the translated NEO-FFI version was assured. The internal consistency of the “Big Five” traits was verified using Cronbach’s alpha resulting in alpha values of α = 0.78 for neuroticism, α = 0.71 for extraversion, α = 0.46 for openness to experience, α = 0.69 for agreeableness, and α = 0.81 for conscientiousness. All traits were satisfactory in term of internal consistency and reliable, with a Cronbach’s alpha greater than 0.6 [68], except for openness to experience, which had an α lower than 0.6. We performed item analysis to try to improve the reliability of the openness to experience trait. The best result was garnered by removing four items; however, Cronbach’s alpha was an unacceptable 0.508, which is consistent with a previous study that indicated the low reliability of the openness to experience measure in Iranian culture, based on the NEO-FFI instrument [52]. For calculating each big traits, we used summed scale [69] in which the score of 12 items related to each trait was summed. Table 1 presents the descriptive statistics of the Big Five traits.

Car habit and intention toward using public transport were measured relatively based on [70] with some modification to adjust the current study. Car habit was measured with four items: “It is unimaginable for me not to use private car for urban and suburban trips,” “So far, I have seldom used public transport,” “Using private car is much more pleasant for me than using another mode of transportation,” and “I feel the best mode of transportation for urban and suburban trips is private car.” Importantly, all four items are expressed positively in relation to car use; however, it would be preferable to have some of the items expressed negatively in relation to car use or favorably in relation to public transport use. Henceforward, further studies are required to test that format for measuring car habits.

The intention was measured using two items as the following: “If the public transportation such as bus and metro become available at IKIA, my future intention to use these public modes to access IKIA is high,” and “I intend to use more public transport in future.”

Cronbach’s alpha for testing internal consistency for car habit and intention toward public transport are 0.67 and 0.61, respectively, which must be greater than 0.60 for a measure to be considered reliable [68]. Therefore, all measures are satisfactory in term of internal consistency.

Respondents were also asked about car access and the number of cars owned by the household. Car access was measured by a single item through five-point Likert scale ranging from never (0) to always (4); “How many times do you have access to a private car?” The calculated mean for car access was 2.88 (Std. Dev. = 1.16), and an average of the number of cars in the household was 1.71 (Std. Dev. = 0.94).

Number of public transport use was also assessed by a single item: “If public modes of transportation such as bus and metro become available at IKIA in future, how many times do you think you will use public transport from each 10 times you have to access IKIA?” with a mean value of 4.84 (Std. Dev. = 3.51). Regarding the use of habit and intention simultaneously in this study, it should be noted that both measures were determined by previous studies to be important in shaping individual behavior [70,71,72], and these studies considered habit and intention concurrently. The intention to use public transport, as mentioned above, was measured by respondents’ answers to two items. The extent of future public transport use was also measured to reflect the forthcoming behavior. However, that the actual behavior was not measured is an unavoidable limitation of this study, owing to the fact that there is no public transportation to IKIA. We recommended that future studies consider the real behavior after the metro line to IKIA is operational. Furthermore, by measuring actual behavior and comparing it with findings of this study, the predictability power of this stated future behavior with actual behavior can be examined. Results of the descriptive statistics of these measures are reported in Table 1.

### 2.3. Methods and Procedures

**Figure 1:**
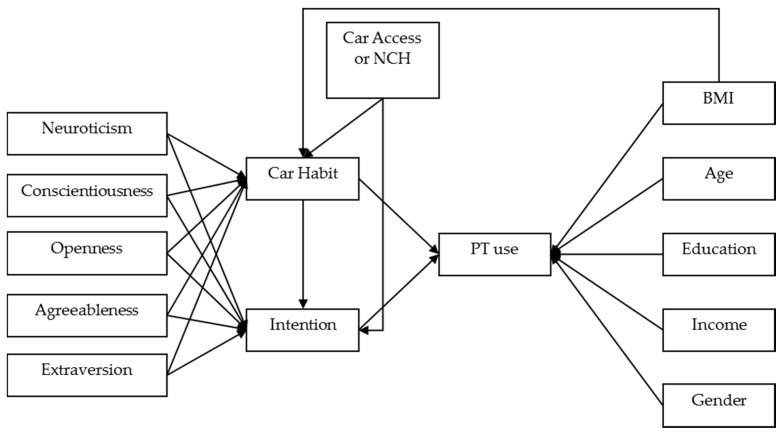
The proposed path model for determining the influence of personality traits through car habit and intention toward public usage on the preference of the number of future public transport usage (PT use), NCH: number of cars in the household, BMI: body mass index.

Structural Equation Modeling (SEM) was used to test the proposed model presented in Figure 1. Personality traits were not found to have a significant direct effect on the number of future public transportation usage. Therefore, in the proposed model, we hypothesized that personality traits indirectly influence the number of public transport use through car habit and intention. Furthermore, it was conjectured that car habit impacts intention. Moreover, we examined separate models to evaluate whether car access or the number of cars in the household could better describe the influence on car habit and intention, on the basis of goodness of fit criteria and significance of relative path direction. Finally, we examined the influence of specific socioeconomic characteristics of individuals such as age, gender, education, and income level on future behavior toward public transport use. In addition, BMI was hypothesized to have significant relation with both car habit and the number of future public transport use. The SEM model of the current study was calibrated using Stata 14 software (StataCorp LLC, College Station, TX, USA).

The goodness of fit statistics which were used in the current study are as follows: the relative or normed chi-square ($\chi^{2}$) test, root mean square error of approximation (RMSEA), the comparative fit index (CFI), Tucker Lewis index (TLI), and standardized root mean residuals (SRMR). It is recommended that a relative chi-square test be less than 5 [73], that RMSEA be less than 0.08 [69], that CFI and TLI be above 0.90 or 0.95 [69], and that SRMR be less than 0.08 [74].

## 3. Results

**Figure 2:**
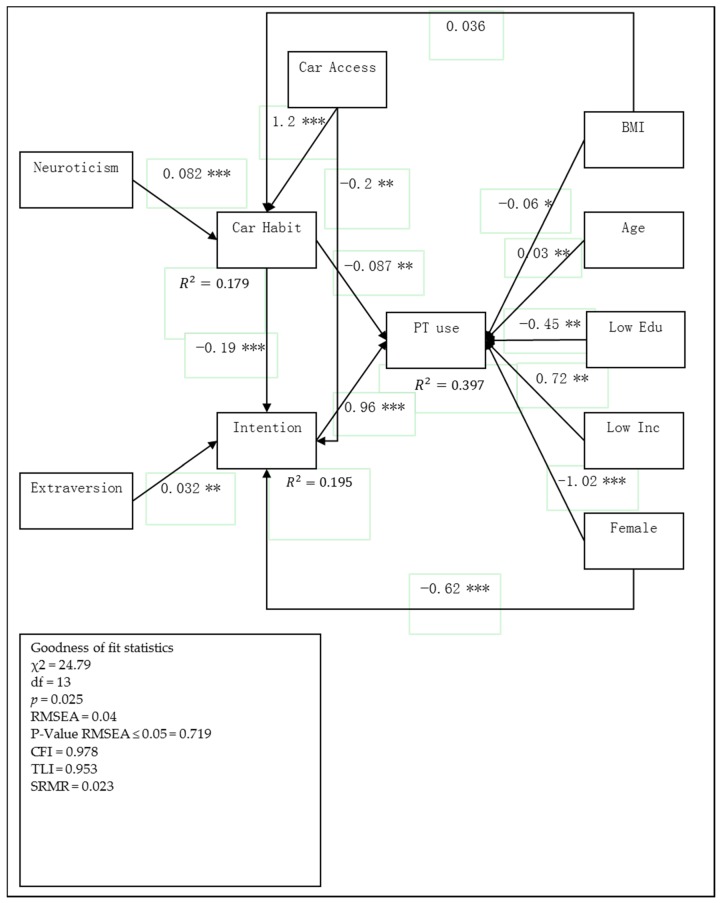
Results of the estimated SEM with car access variable effecting on car habit and intention toward public usage (* *p* ≤ 0.05; ** *p* ≤ 0.01; *** *p* ≤ 0.001); the sample size is equal to 557. Modified from the proposed model. PT use: the number of future public transport usage.

**Figure 3:**
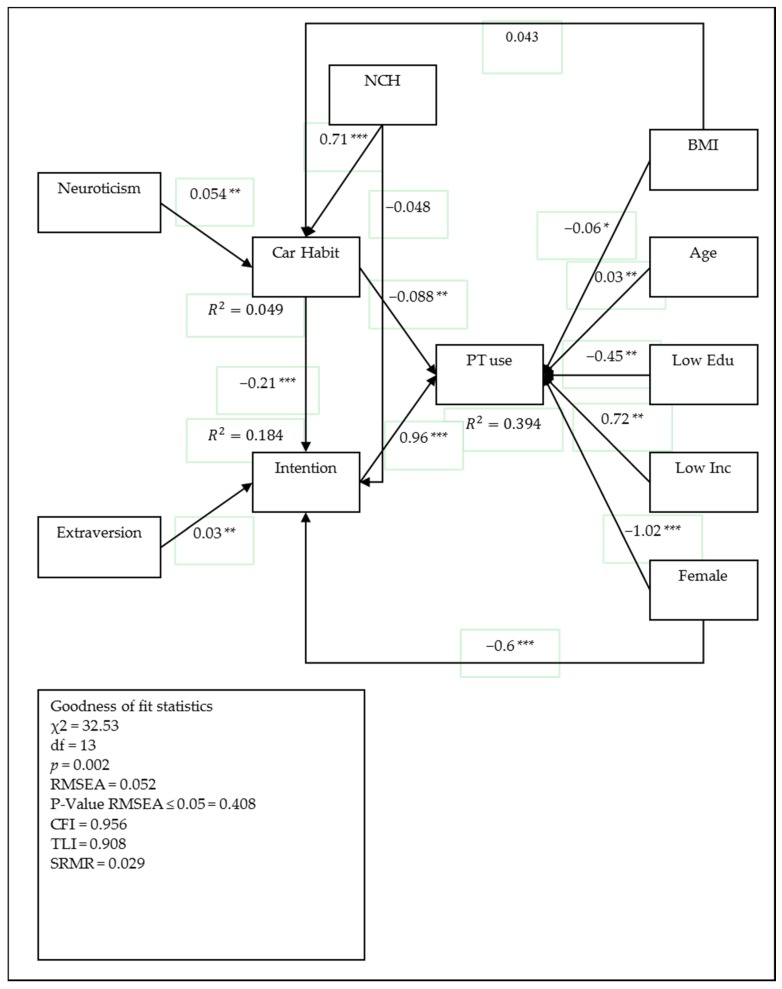
Results of the estimated SEM with the number of cars in household (NCH) variable effecting on car habit and intention toward public usage (* *p* ≤ 0.05; ** *p* ≤ 0.01; *** *p* ≤ 0.001), the sample size is equal to 557. Modified from proposed model. PT use: the number of future public transport usage.

Figure 2 depicts the SEM model with car access variable affecting car habit and intention, whereas Figure 3 represents the model in which car access was replaced with the number of cars in household (NCH). Both models represent the same results which reveal the positive effect of car access and NCH on car habit and negative effects on intention toward using public transport. However, the model with NCH variable does not have a significant impact on intention and the model with car access demonstrates the better goodness of fit. These results indicate the usefulness of car access over NCH in transportation studies. Hence, it is recommended to consider car access in future studies. Additionally, the model with NCH explained 39.4% of the variance in the number of future public transport use by NCH, intention, and socio-demographic variables. In comparison, 18.44% of the variance in intention was explained by extraversion, car habit, and gender variables. Only 4.9% of the variance in car habit was explained by neuroticism, NCH and BMI variables in which the influence of BMI on car habit was not significant.

On the other hand, the model with car access had a good fit to the data and 39.7% of the variance in the number of future public transport use was explained by car access, intention, and socio-demographic variables. In addition, 19.50% of the variance of intention was explained by extraversion, car habit, and gender variables; while 17.90% of the variance in car habit was explained by neuroticism, car access, and BMI variables. In the following section, only results of the model with car access variable which has good fit indices are reported and will be discussed.

As demonstrated in the previous section of the paper, the direct effect of personality traits on the number of public transport usage in future does not show any significant results. Therefore, the paper seeks the relation though car habit and intention. Results of SEM analysis show that three of the big personality traits, namely conscientiousness, openness to experience, and agreeableness do not have any significant relation to car habit and intention, and have therefore been dropped from the model. Interestingly, neuroticism, which shows the individuals with high values on this measure are more likely to be anxious and prone to psychological distress [75], had a significant positive effect on car habits. Furthermore, a high value for extraversion indicates that an individual is lively, cheerful, and sociable [57]; this measure was found to positively affect intention toward public transport use.

Neuroticism, with the total effect of −0.022 on future public transport use, has a negative association with public transport use. In contrast, extraversion had a total effect of 0.031, indicating a positive association with future public transport use. Therefore, individuals who obtain high values on neuroticism are less likely to use public modes of transportation in the future, whereas those who have high values on extraversion are more willing to use public modes of transportation more frequently.

Moreover, Socio-demographic variables were found to significantly influence the number of future public transport use; Increasing BMI, having lower education, and being female were negatively associated with the number of public transport use. The effect of the age variable shows that it has a positive relation to future public transport use by increasing the age of individuals. Low-income individuals also show a positive relation in using a public mode of transport to IKIA. Finally, the results may shed some ideas for increased understanding of the psychological structures underlying transport use and for developing more effective and adaptive policy, which is discussed in the next section.

## 4. Discussion

This study seeks to find the relation between personality traits, habit, and intention as they pertain to future public transport use, and the findings of this study are expected to support decision-makers in developing more adaptive strategies and promoting the use of sustainable modes of transportation.

Consistent with previous studies [39], we found that car habit negatively influences both intention toward public transport use and the number of public usage in the future. As expected, intention was positively related to future public transport usage. It has been argued that habit could have the strongest role on behavior when the circumstances remain stable [32] and in a stable condition of context and information stream [76]. When habit was strong, no intention-behavior relation existed [37]. Therefore, policy makers should strive to change habits with appropriate information to strengthen the intention–behavior relation and apply good promotional offers to break the car habit choice [77] as soft measures or interventions for more public transport use has proven to be promising [78].

Furthermore, in order to effectively plan for actively encouraging public transport use, an in-depth understanding of how personality traits influence individuals’ behavior in this subject area is crucial. Moreover, this study confirms the underlying assumptions of the Theory of Planned Behavior, which assumes that personality traits have little impact on behavior. However, a little knowledge of each group of individuals based on their personality traits may help in producing more effective policies regarding increasing public transportation use or discouraging car use. Another study indicated limited success of interventions to reduce car use [79], and beliefs that underpin TPB-specified cognitions that are not as well understood were investigated. Therefore, for increased understanding of the cognitive underpinnings of the TPB, various psychological factors must be tested, which could enhance the predictability power of this theory.

A previous study showed that car use was determined by intention and habit, while public transport use was influenced solely by intention [71]; our study contradicts this by finding that public transport use is under influenced by both habit and intention.

Additionally, the habit–discontinuity hypothesis states that behavior is more deliberately disrupted when it comes in the context of a life change [80]. Correspondingly, when the metro line to IKIA eventually opens, which is currently under construction, it may act as a disruptor of current car use habits. The window of opportunity for implementing effective interventions is considered to be open three months after a context change [81]; hence, transportation planners should be aware of the importance of the three-month period following the opening the IKIA metro line. Clearly, this would be an important time to make an intervention to promote and motivate individuals to utilize public transport.

Regarding including the car access variable, a previous study that investigated the impact of habit and car access on student behavior uncovered that habit was the strongest factor influencing behavior, while car access only significantly influenced habits (rather than having a direct effect on actual behavior [35]). Our study is consistent with this earlier study. Therefore, these results have value for transportation modelers to use car access instead of the customary variable of number of vehicle owned in the household.

The results for individuals’ BMI score are in line with previous studies that indicated that public or active transport commuters had significantly lower BMI numbers [40,43,82]. Furthermore, lifestyle habits were previously determined to have a positive correlation with BMI [42], and our study also showed a positive relationship between these two factors. In our study, however, this relation is not statistically significant.

However, higher age and having lower income level were positively related to the number of public transport use. Concerning the result of the age variable, it is contrary to the study of elderly air passengers [83], which may be due to cultural differences.

The modification fit indices were used to test additional possible paths for improving the model fit to the data and were found that gender has a significant negative effect on intention. Females have also been shown to have less intention toward public transport use. This is in contrast with the study of examining gender differences in the willingness to reduce car use [84], which indicated women are more willing to reduce car use because of their stronger ecological norms and weaker car habits. This controversial result may be attributed to cultural differences and the type of destination access (i.e., the airport in the current study was located outside the city boundaries).

Moreover, the findings of the current study which investigated the effect of personality traits can suggest new ways for determining future public transport use as a sustainable mode. This expanded understanding reveals that apart from the significant objective attribute of such as travel time and travel cost as conventional predictors of using public transportation modes, individuals with distinct personality traits behave differently regarding future use of sustainable modes of transportation (public transport use).

Because travel behavior is thought be better understood if underlying psychological factors are known [85], our study attempts to add personality traits to this subject area. Therefore, it would be worthwhile to measure personality traits in relation to public transportation use in order to bring new perspectives for a more precise understanding of sustainable behaviors. Such psychological variables may be helpful in predicting the use of transportation modes. Furthermore, socioeconomic status (SES) differences in Iran can be significant, and they may have more influence on public transport use than in other countries, which aim to minimize these differences [39]. As a result, this study can offer a new perspective from the context of a country with highly stratified SES differences on public transport use.

This study is limited by the data that focused purely on accessing IKIA and its distinct respondents. IKIA passengers are somewhat individualized, because this airport mostly serves international flights, and there is no public transit service currently for this airport.

However, a number of other limitations also affect this study. One is that the questionnaire is a part of a longer survey which aimed at capturing several aspects of travel behavior, its length may challenge respondents. In addition, airport changes occur frequently and, though travelers may have free time before flights, they worry about getting boarding passes and other required travel documents. Moreover, passengers who arrive late to the airport may be excluded from the study because they may not have sufficient time to complete the questionnaire. These sample biases may limit the generalizability of findings to the international air traveling population of Iran.

In addition, traveling to the airport is not a commuting trip, and the behavior might be different. The generalizability may be limited to airport access in developing, religious countries with airports such as those in Iran. Therefore, the generalizability to other cultures may be limited. Furthermore, this study makes use of data from a survey specifically designed to analyze transportation mode choice based on discrete choice modeling; therefore, the availability of data was limited to conducting TPB in this study.

## 5. Conclusions and Future Directions

It has been argued that the transportation mode choice could be explained by individuals’ latent traits [86]. The effect of personality traits, especially based on the well-known five-factor model on transport use, is unclear. As such, the aim of the present study was to assess the role of personality traits, habit, and intention in determining sustainable public transport use in the future. To the best of our knowledge, this study is the first attempt in considering the probable association of personality traits with future public transport use mainly using a sample of airport passengers. A case study of accessing toward an airport was used for this purpose at IKIA. The results suggest an indirect effect of personality traits on future public usage through car habit and intention, while a direct effect was not supported by the data. A significant, direct link of personality traits to public transport use was not identified, but that may be attributed to the summed scale measures used in this study. Therefore, this study explores the mediating effect of habit and intention in linking personality traits to public transport use in the future.

The results of this paper may also aid in the understanding of subjective factors, especially personality traits, habit, and intention, and the knowledge gained from the results have importance for psychologists, transportation planners and policy makers in understanding different behaviors of individuals based on their personality traits. Additionally, the results suggest that the influence of personality traits is minor, but personality traits may have a significant effect on predicting public transport use in the future. However, this minor influence may be attributed to summed scale measures used throughout this study, and it would be recommended that future studies use the most advanced and accurate analysis with latent variables to identify structures under the influence of these subjective and cognitive factors on transportation mode use.

In the light of our findings, future studies can replicate the study in different countries and cultures that may behave differently. Additionally, as the current study is based on observable variables (summed scales), future efforts can be made by conducting SEM based on latent variables through confirmatory factor analysis. Furthermore, the endogenous variable of future public transport use did not measure the actual behavior but the stated situation of future behavior because currently there is no public transport available at IKIA. Future attempts can employ a survey in two stages: the present situation and when public mode becomes available. Furthermore, implementing the Theory of Planned Behavior is recommended to more accurately explain the variation in intention and model fit, and thus the mode use behavior by considering attitude, subjective norm, and perceived behavioral control. Additionally, the comprehensive action determination model can be implemented for a better understanding of travel behavior; the model showed that car access directly influences car habit and transport mode use, while it had an indirect effect through perceived behavioral control on intention [70]. Transport mode shift is a complicated process, and future research must consider different and more comprehensive socio-psychological variables beside typical socio-demographic and transportation mode attributes. Therefore, we recommend that future research uses the more comprehensive NEO-PIR instrument to assess the relation of the openness to experience trait in Iran. Additionally, much attention is needed to understand the development of car use habits during the life stages and contextual factors that affect travel mode choices [87]. Moreover, researchers can focus on developing a tool to measure habit and intention more precisely.

## Figures and Tables

**Table 1:** Descriptive statistics of constructs measured based on summed scale, sample size: 557.

Construct	Min	Max	Mean	Stdv
Neuroticism	0	39	18.37	6.93
Conscientiousness	16	48	36.17	5.72
Openness to experience	15	42	27.99	4.76
Agreeableness	13	48	32.13	5.57
Extraversion	11	46	29.92	5.54
Car habit	0	16	7.97	3.43
Intention	0	8	4.71	1.89
